# Supplementary material for: Causal Relationship between Aging and Anorexia Nervosa: A White-Matter-Microstructure-Mediated Mendelian Randomization Analysis
Source: Biomedicines. 2024 Aug 16;12(8):1874. doi: 10.3390/biomedicines12081874 (PMC11351342; doi:10.3390/biomedicines12081874)
Supplement: Supplementary file 1 [file biomedicines-12-01874-s001.zip › Supplementary Figure/Figure S8/Leaveoneout plot of MR analysis for TL to ubm-b-2015.pdf]

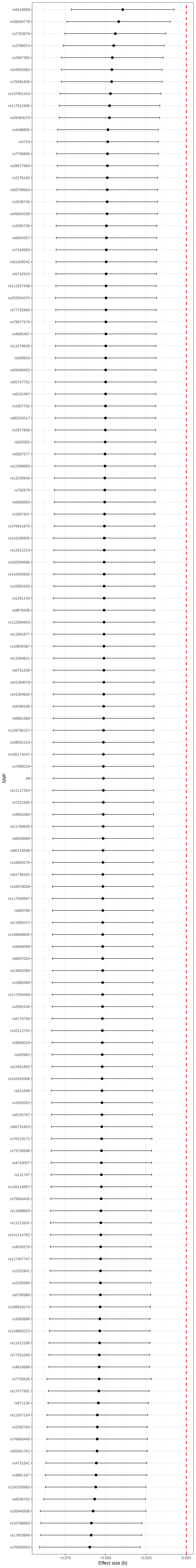
rs9419958
rs35640778
rs2763979
rs3785074
rs2967355
rs34550383
rs78491606
rs137901416
rs117512405
rs28363070
rs4498805
rs4724
rs7790856
rs28577594
rs2276182
rs56799554
rs2538745
rs45604339
rs2056726
rs6054257
rs7164950
rs61405042
rs5742915
rs111527438
rs202034370
rs77732866
rs79977579
rs4695407
rs11579626
rs939916
rs59409453
rs55747751
rs8102497
rs1907702
rs80324517
rs2977608
rs932002
rs6587577
rs12369950
rs13230646
rs762679
rs6669563
rs1957937
rs376641875
rs143190905
rs12412214
rs182059586
rs144204502
rs10905255
rs1291143
rs9878436
rs112394943
rs11991877
rs10845387
rs11584821
rs6751209
rs41269079
rs41304832
rs9398196
rs6881568
rs139795227
rs28502153
rs185174247
rs7099229
All
rs11117354
rs7221585
rs9955360
rs11769630
rs6659669
rs80116508
rs10840270
rs81748181
rs16978028
rs117630647
rs869785
rs11085072
rs139669835
rs9940099
rs6007020
rs13062095
rs1985369
rs117034449
rs2555104
rs6776756
rs10112752
rs9600019
rs450962
rs12451892
rs142426306
rs611646
rs1003322
rs8105767
rs66731853
rs76219171
rs73730598
rs4743037
rs131797
rs145114957
rs75664430
rs11699829
rs11212631
rs141214782
rs4530278
rs117407747
rs1332941
rs2230590
rs6790988
rs188918174
rs3093888
rs116863223
rs11412296
rs77231040
rs4616688
rs7705526
rs17677991
rs871134
rs11557154
rs2282764
rs76666449
rs56061761
rs4731541
rs3891167
rs150150565
rs6536702
rs35446936
rs10766683
rs17803849
rs76065543
SNP
-0.075
-0.050
-0.025
0.000
Effect size (b)
